# Dissociation Dynamics of XPC-RAD23B from Damaged DNA Is a Determining Factor of NER Efficiency

**DOI:** 10.1371/journal.pone.0157784

**Published:** 2016-06-21

**Authors:** Benjamin Hilton, Sathyaraj Gopal, Lifang Xu, Sharmistha Mazumder, Phillip R. Musich, Bongsup P. Cho, Yue Zou

**Affiliations:** 1 Department of Biomedical Sciences, Quillen College of Medicine, East Tennessee State University, Johnson City, Tennessee, 37614, United States of America; 2 Department of Biomedical and Pharmaceutical Sciences, College of Pharmacy, University of Rhode Island, Kingston, Rhode Island, 02881, United States of America; University of South Alabama Mitchell Cancer Institute, UNITED STATES

## Abstract

XPC-RAD23B (XPC) plays a critical role in human nucleotide excision repair (hNER) as this complex recognizes DNA adducts to initiate NER. To determine the mutagenic potential of structurally different bulky DNA damages, various studies have been conducted to define the correlation of XPC-DNA damage equilibrium binding affinity with NER efficiency. However, little is known about the effects of XPC-DNA damage recognition kinetics on hNER. Although association of XPC is important, our current work shows that the XPC-DNA dissociation rate also plays a pivotal role in achieving NER efficiency. We characterized for the first time the binding of XPC to mono- *versus* di-AAF-modified sequences by using the real time monitoring surface plasmon resonance technique. Strikingly, the half-life (t_1/2_ or the retention time of XPC in association with damaged DNA) shares an inverse relationship with NER efficiency. This is particularly true when XPC remained bound to clustered adducts for a much longer period of time as compared to mono-adducts. Our results suggest that XPC dissociation from the damage site could become a rate-limiting step in NER of certain types of DNA adducts, leading to repression of NER.

## Introduction

The human genome is constantly under assault from exogenous and endogenous causes of DNA damage. The formation and propagation of the resulting adducts can be particularly destructive when these mutations occur within tumor suppressing genes, leading to tumorigenesis [1–4]. Consequently, human cells have several effective DNA repair pathways to protect against the plethora of genotoxic bombardments to the genome [5]; however, the mechanism by which damage-recognition proteins distinguish damage sites remains uncertain. Mutations that arise in genes associated with the nucleotide excision repair pathway (NER) result in a multitude of genetic disorders such as *xeroderma pigmentosum*, which is characterized by sensitivity to sunlight and, ultimately, the development of carcinomas [6].

NER is utilized to remove primarily bulky adducts, plus cross-links, and various other lesions [7–9]. NER is either associated with transcription in transcription-coupled repair (TCR) or is independent of transcription in global genome repair (GGR). GGR in *Escherichia coli* consists primarily of a collaborative effort of three proteins that both recognize and incise damaged bases: UvrA, UvrB, and UvrC [10]. Two UvrA molecules associate and then form a trimeric complex with UvrB. This trimeric complex is thought to be the DNA damage sensor. UvrA facilitates UvrB binding and positions UvrB to confirm the existence of a damage site. Once UvrB is in the correct position, UvrA utilizes its ATPase activity to dissociate from the preincision complex. UvrB then recruits UvrC endonuclease, which incises the damaged DNA strand by 3’ and 5’ cleavages flanking the damage site [11–14]. In human GGR the UvrA_2_B functional equivalent is *Xeroderma pigmentosum* group C (XPC) in complex with RAD23B (XPC-RAD23B, henceforth XPC). The XPC complex acts in the DNA damage recognition step, thus initiating GGR [15]. XPC has been shown to bind at the site of many types of damage *in vitro* and in UV-treated cells arrives at damage sites before other NER factors [9,16–18]. Once at the damage site XPC recruits the multi-subunit transcription factor TFIIH, including the helicase subunits of XPB and XPD, followed by XPA for damage confirmation, fork binding and subsequent recruitment of replication protein A (RPA) for single-stranded DNA (ssDNA) stabilization, and XPG and XPF-ERCC1 for the dual incisions [19–22].

Crystal structures of the yeast XPC-RAD23B ortholog, Rad4-Rad23, in association with undamaged or damaged DNA revealed a mechanism by which XPC hops along DNA until a thermodynamically stable recognition complex is formed, which effectively distinguishes damaged from non-damage sites [23,24]. Further studies have supported this hypothesis by suggesting that residence time of XPC on damages may play a role in the relationship between XPC binding and NER efficiency [25,26]. Binding affinity of XPC at the damage site has been suggested to be the rate-limiting step for NER [25,27,28]. Although various efforts have been made to correlate the equilibrium binding of damage recognition to overall NER efficiency, little is known about the role of the kinetics of damage recognition in the NER process.

**Fig 1:**
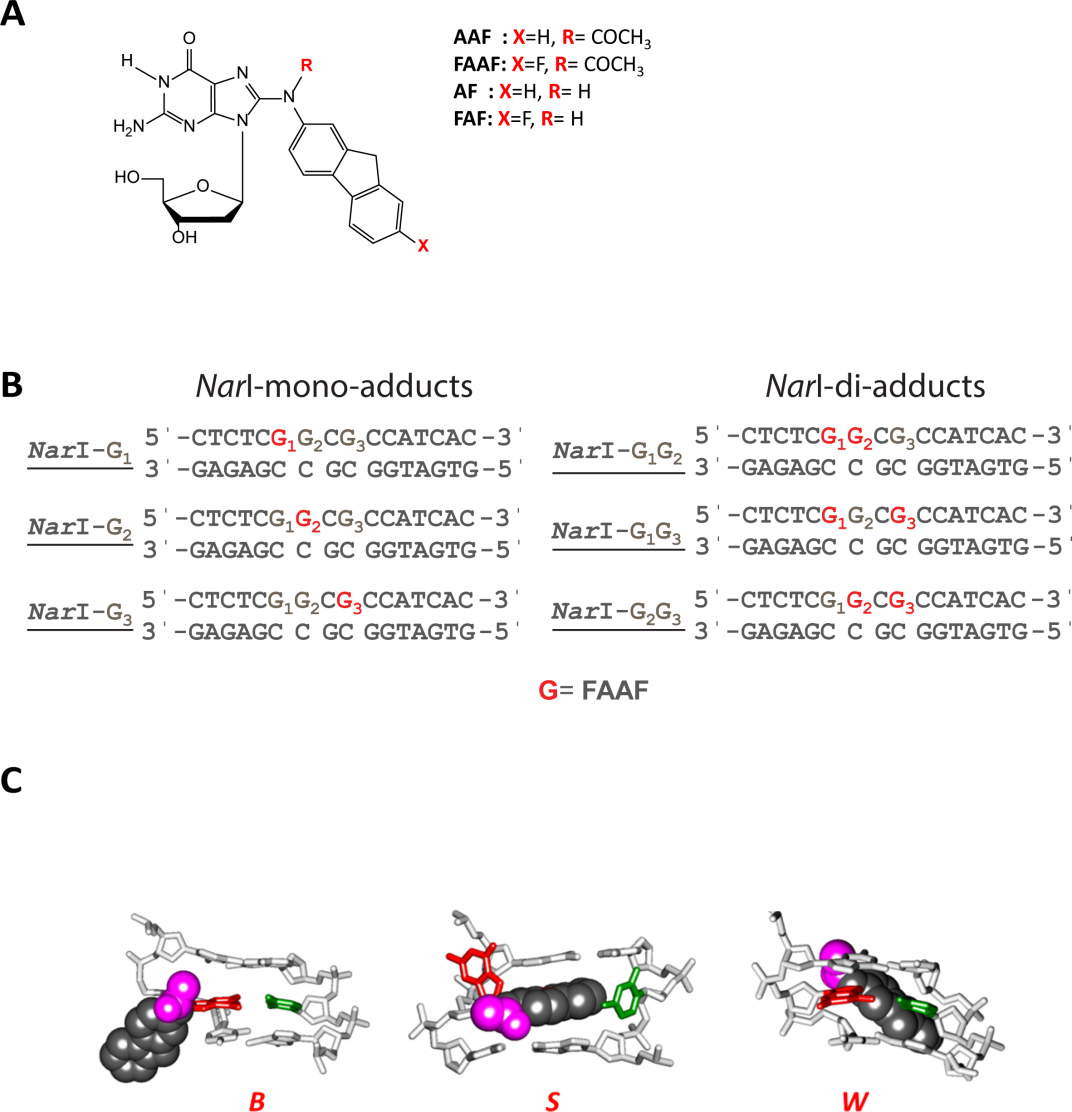
Adduct structures and sequences. (A) Structure of AAF [*N*-(2’-deoxyguanosin-8-yl)-2-acetylaminofluorene], AF [*N*-(2’-deoxyguanosin-8-yl)-2-aminofluorene] and fluoro models, FAAF [*N*-(2-deoxyguanosin-8-yl)-7-fluoro-2-acetylaminofluorene], FAF [*N*-(2’-deoxyguanosin-8-yl)-7-fluoro-2-aminofluorene]; (B) Fully-paired 16-mer duplexes containing the central *Nar*I sequence (CGGCGCC) used in SPR, EMSA and *in vitro* NER constructs illustrating the placement of the adducted bases at G_1_, G_2_, and G_3_ positions; (C) Major groove views of the B-, S-, and W-conformers of AAF. Modified-dG (red), dC (green) opposite the lesion site (orphaned C), fluorene (grey CPK), *N*-acetyl (magenta).

Arylamines and heterocyclic amines are notorious environmental carcinogens. The DNA adduct-forming arylamines can be found naturally in the environment, in addition to a number of unnatural sources such as cigarette smoke and hair dyes. Heterocyclic amines are most notably abundant in meat that has been cooked at high temperatures. It is inevitable that a person will be exposed to one or both of these carcinogens in his/her lifetime. Each of these mutagens has been documented to cause many types of cancer, such as breast, liver, and bladder, to name a few [2]. Metabolic activation of these amines *in vivo* produces C8-substituted dG as the major bulky DNA adduct [29]. A well-known example is the human bladder carcinogen 4-aminobiphenyl [30]. The prototype environmental arylamine 2-aminofluorene produces two major DNA adducts *via in vivo* activation: N-(2′-deoxyguanosin-8-yl)-2-aminofluorene (AF) and N-(2′-deoxyguanosin-8-yl)-2-acetylaminofluorene (AAF) (Fig 1A) [31]. Their fluorine derivatives FAF and FAAF have been used extensively as ^19^F NMR conformational models for these bulky arylamine lesions [32–34]. Conformational studies have shown that FAF in a fully paired duplex DNA can adopt an equilibrium between two prototype conformers, while the N-acetylated FAAF exhibits an additional conformation due to a single bulky acetyl group on the central nitrogen. This equilibrium exists between major groove binding anti B-type conformers, base-displaced *syn* stacked S conformers, and minor groove binding of the *syn* adduct wedge (W) conformer (Fig 1C) [33,35]. Usually, the damage produced is a single mono-adduct: however, cluster di-adducts can form *in vivo*, though less frequently than mono-adducts as discussed previously [36–38]. Past work has shown that adduct conformation is strongly dependent on the flanking sequence, which modulates mutational and repair outcomes [27,39–41]. One such sequence is the mutational hotspot known as the *Nar*I sequence (5’-… CG_1_G_2_CG_3_CC… -3’) (Fig 1B), which has been extensively studied [33,42].

The reparability of adducts in the *Nar*I sequence has been tested in both the *E*. *coli* UvrABC and human endonuclease systems and were found to be sequence dependent [27,33,43]. In addition, different repair efficiencies of the same lesions were observed between the two systems [27,33]. Furthermore, recent work has attempted to correlate the binding affinities of repair proteins with adduct excision or NER efficiency [25–27]. Yeo and colleagues, implementing electrophoretic mobility shift (EMSA) and dual-incision assays, concluded that increased DNA thermodynamic destabilization, XPC-RAD23B binding, and overall NER efficiency of AAF adducts are directly correlated [25]. In contrast, Mu *et al*. showed that NER efficiencies of the same AAF lesions are correlated with greater extents of base sequence-dependent local untwisting and minor groove opening together with weaker stacking interactions [27]. Lee *et al*. have found minimal differences in XPC binding affinities of lesions derived from bulky polycyclic aromatic hydrocarbons while observing dramatic differences in NER efficiency [26]. These three individual reports employed different bulky adducts in their studies; however, Shell *et al*. demonstrate that XPC acts as a general sensor for DNA damage, with a preferential binding to damage sites, but concluded that lesion identity is not a determinant of XPC binding affinity [44].

In the present study, we analyzed the kinetic aspects of *E*. *coli* UvrA_2_ and human XPC protein interactions with AAF adducts in the *Nar*I sequence context by surface plasmon resonance (SPR) analysis and defined the relationship with NER. SPR has the significant advantage over other methods designed to observe protein-DNA interactions in that the interaction can be observed in a dynamic real-time environment, much closer to native conditions. We show that at lesion clusters the kinetic off-rate of XPC has an inverse correlation to repair efficiency. In other words, the *t*_*1/2*_ (time required for 50% of the bound XPC to dissociate from the DNA, *t*_*1/2*_ (s) = ln(2)/k_d_,) of the damage recognition complex inversely correlates to NER efficiency. This work reveals the significance of the dynamics of XPC recognition of conformationally diverse DNA adducts in NER. Here, we describe a new model for XPC activation of NER where the off-rate of XPC from the lesion site, particularly in the case of clustered lesions, is the rate-limiting step of NER, and propose applying this finding to design a more efficiently targeted approach to cancer therapy.

## Materials and Methods

### Caution

2-Aminofluorene derivatives are mutagens and suspected human carcinogens and, therefore, must be handled with caution.

Crude desalted oligodeoxynucleotides (1 μmol) were purchased from Operon (Eurofin, Huntsville, AL) and purified by reverse phase HPLC. All HPLC solvents were purchased from Fisher Inc. (Pittsburgh, PA).

### Substrate preparation and characterization

Modified duplexes of 55 bp DNA substrates containing mono-FAAF and di-FAAF adducts in the *Nar*I sequence context were constructed as previously described [32,35,38]. The HPLC purification system consisted of a Hitachi EZChrome Elite system with an L2450 diode array as a detector and a Clarity column (10 mm × 150 mm, 3 μm) (Phenomenex, Torrance, CA). Purification of oligodeoxynucleotides was carried out using a 20 min linear gradient mobile phase system from 5 to 20% (v/v) acetonitrile with 100 mM ammonium acetate buffer (pH 6.5) at a flow rate of 2.5 mL/min.

### Preparation of arylamine-modified template

The modified 55-mer biotinylated DNA templates were prepared according to published procedures [45–47]. Mono- and di-adduct oligodeoxynucleotides in the *Nar*I modified strand were purified by HPLC (described above) and characterized by Shimazdu Axima MALDI-TOF mass spectrometry as previously reported [38] (Fig 1 and S3 Fig). 5’-Biotinylated 55-mer (1 OD) was annealed with 55-mer complementary strand (1.05 ODs) in 1x HBS-EP^+^ buffer for 5 min at 95°C. Identical unmodified duplexes were concurrently prepared as controls. The annealed oligodeoxynucleotides then were used for SPR experiments.

### Oligonucleotide sequence used for surface plasmon resonance

5’-biotin-CCACTCCTATCCACCATCCATCTTACTCTCG_1_G_2_CG_3_CCATCACCACTCACCACCACA-3’

3’-GGTGAGGATAGGTGGTAGGTAGAATGAGAGC C GC GGTAGTGGTGAGTGGTGGTGT-5’

G_1_, G_2_, and/or G_3_: dG or dG-FAAF

### Purification of XPC-RAD23B protein complex

XPC-RAD23B protein was prepared from Sf21 insect cells infected with recombinant baculovirus expressing XPC and RAD23B proteins (graciously provided by A. Sancar, University of North Carolina, Chapel Hill). The XPC-RAD23B complex was purified as described previously [48,49]. Protein concentration was determined using the Bio-Rad protein assay. Following purification by size-exclusion chromatography, SDS-PAGE (10%) and Western blotting confirmed the purity of the XPC-RAD23B complex.

### Immobilization of streptavidin on CM5 chip and DNA coating

SPR measurements were conducted with a Biacore T200 (GE Healthcare). Streptavidin (SA) was immobilized on a CM5 dextran chip using an amine-coupling method [45,47]. Four flow cells were immobilized with streptavidin amine to ~2,200 resonance units (RU). Flow cell 1 was used as a reference. Before the coating of biotinylated DNA templates over SA, the surface was washed with 50 mM NaOH five times, each with 60s pulses at 100 μl/min to remove any free SA until the change in response units was below 5 RU. The surface was further injected 3–4 times with HBS-P+ running buffer (10 mM HEPES, 150 mM NaCl, 0.05% non-ionic surfactant P20) to remove any residual NaOH in the microfluidics path and to stabilize the surface. Biotinylated unmodified and various FAAF-modified DNA duplexes (0.025 nM) were injected at 100 μl/min for 240–300s over the flow cells 2, 3, or 4 to achieve 2–5 RU relative to flow cell 1, which was a blank reference. Any unbound DNA was washed away with running buffer.

### Kinetics analysis

The binding kinetics for the interaction of UvrA or XPC with DNA was determined by injecting the UvrA (0–500 nM) or XPC (0–5 nM) in HBS-P+ running buffer containing 5 mM MgCl_2_, 1 mM DTT and BSA (100 μg/mL). The flow rate was 100 μL/min for 30 s followed by dissociation for 60 s. The DNA surface was conditioned by a sequential injection involving 1x HBS-P+ running buffer, 3x HBS-P+ buffer, and 4x HBS-P+ buffer (prior to addition of enzyme). The surface was regenerated with a 30 s injection of 0.05% SDS with a flow rate of 100 μL/min, followed by a wash with HBS-P+ running buffer. Experiments were repeated 3x with duplex injections of the indicated concentrations. UvrA binding studies were performed in the presence of ATP (0.5 mM). All SPR sensograms were double referenced and fitted using a simple 1:1 Langmuir model (S1 Fig). Processing included zeroing and cropping data, aligning injection times, fitting of binding curves and off-rate analysis. The equilibrium dissociation constant (K_D_) for ternary systems was calculated using the steady-state affinity analysis in the BIA-Evaluation software package v2.0 provided by the manufacturer, General Electric. The average of the data (with standard deviation) of K_D_, k_a_, and k_d_ is shown in Tables 1 and 2. The Scrubber software package (BioLogic Software) was used to process off-rate analysis of raw XPC-H23B SPR binding sensograms. Curve fittings were not ideal for certain UvrA data (S2 Fig), which affected the reliability of rate constants (see Results).

**Table 1:** Correlation of XPC-RAD23B binding and dissociation parameters, melting temperature, and hNER efficiencies of FAAF-modified *Nar*I substrates.

*Nar*I-FAAF	SPR (K_D_) (M)	SPR (k_a_) (M^-1^s^-1^)	SPR (k_d_) (s^-1^)	t1/2 (s)	T_m_(°C)(ΔT_m_)	hNER
CCG_1_*G_2_CG_3_CC (G_1_-mono)	1.8 (±0.02) x 10^−9^	1.1 (±0.01) x 10^7^	1.9 (±0.02) x 10^−2^	24 (±0.5)	68.6 (-5.3)	1 (±0.14)
CCG_1_G_2_*CG_3_CC (G_2_-mono)	0.9 (±0.01) x 10^−9^	6.9 (±0.17) x 10^7^	6.3 (±0.16) x 10^−2^	44 (±0.4)	66.0 (-7.9)	0.69 (±0.03)
CCG_1_G_2_CG_3_*CC (G_3_-mono)	1.1 (±0.01) x 10^−9^	3.7 (±0.07) x 10^7^	4.2 (±0.08) x 10^−2^	38 (±0.1)	65.6 (-8.3)	0.65 (±0.06)
CCG_1_*G_2_*CG_3_CC (G_1_G_2_-di)	7.6 (±0.26) x 10^−11^	1.0 (±0.008) x 10^8^	2.5 (±0.01) x 10^−3^	102 (±1.3)	60.6 (-10)	0.69 (±0.08)
CCG_1_*G_2_CG_3_*CC (G_1_G_3_-di)	1.6 (±0.76) x 10^−11^	1.4 (±0.008) x 10^8^	6.7 (±0.05) x 10^−3^	282 (±2.9)	56.5 (-14.1)	0.30 (±0.05)
CCG_1_G_2_*CG_3_*CC (G_2_G_3_-di)	0.42 (±0.34) x 10^−11^	1.4 (±0.01) x 10^8^	1.4 (±0.01) x 10^−3^	492 (±4.5)	52.7 (-17.9)	0.12 (±0.02)

**Fig 2:**
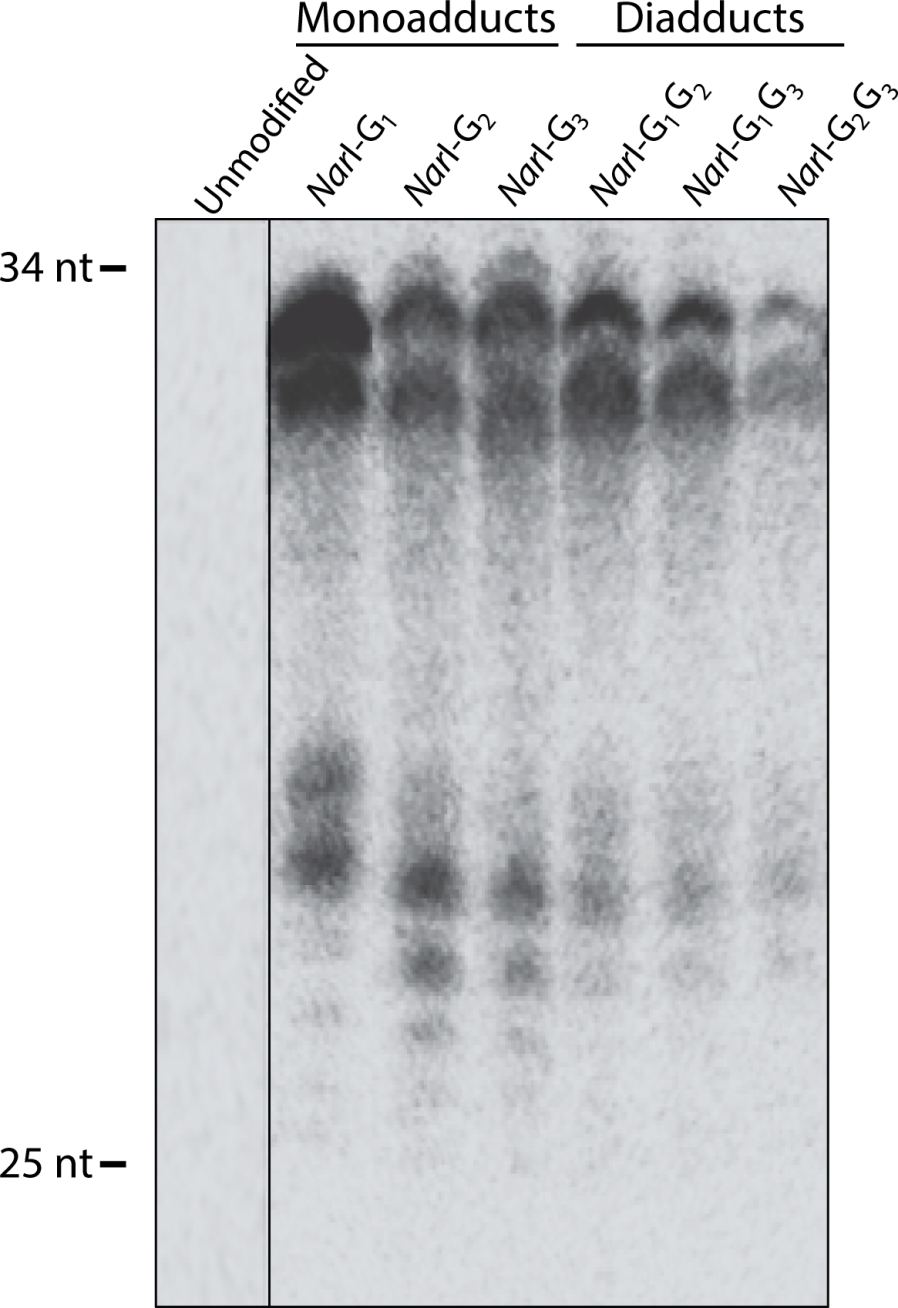
NER dual incision at adducts in the *Nar*I sequence in the human NER system. Plasmids containing site-specific mono-FAAF (lanes G_1_, G_2_, G_3_) or di-FAAF adducts (lanes G_1_G_2_, G_2_G_3_, G_1_G_3_), were incubated with HeLa whole-cell extracts. Detection of excision products was monitored by 3’-end-labeling using a complementary oligonucleotide containing a 5’-GGGG base overhang. The reaction products were resolved on a 12% denaturing polyacrylamide gel run under constant current. The range of excision products is indicated on the left of the gel.

There is an inverse relationship between off-rate kinetics and human NER of the di-adducted dG-FAAF substrates. SPR (k_a_), SPR (k_d_) and SPR (K_D_) are the association rate (k_a_), dissociation rate (k_d_) and equilibrium dissociation constant (K_D_) values determined by SPR analysis of the interaction of XPC with mono- and di-adducts and t_1/2_ (s) is the calculated half-life of the protein-DNA complex. The T_m_(°C)ΔT_m_ data are the thermodynamic stability as previously reported [33,38]. The hNER efficiency is relative to the data displayed in Fig 2.

**Table 2:** Binding and dissociation parameters of UvrA_2_ binding to FAAF-modified *Nar*I substrates.

*Nar*I-FAAF	SPR (K_D_) (M)	SPR (k_a_) (M^-1^s^-1^)	SPR (k_d_) (s^-1^)
CCG_1_*G_2_CG_3_CC (G_1_-mono)	1.3 (±0.01) x 10^−9^	4.9 (±0.05) x 10^6^	0.6 (±0.005) x 10^−2^
CCG_1_G_2_*CG_3_CC (G_2_-mono)	2.4 (±0.04) x 10^−9^	1.8 (±0.005) x 10^6^	0.4 (±0.002) x 10^−2^
CCG_1_G_2_CG_3_*CC (G_3_-mono)	3.9 (±0.05) x 10^−9^	1.8 (±0.004) x 10^6^	0.7 (±0.002) x 10^−2^
CCG_1_*G_2_*CG_3_CC (G_1_G_2_-di)	4.9 (±0.46) x 10^−10^	6.2 (±0.06) x 10^6^	0.3 (±0.02) x 10^−2^
CCG_1_*G_2_CG_3_*CC (G_1_G_3_-di)	2.7 (±0.46) x 10^−10^	5.2 (±0.01) x 10^6^	0.1 (±0.01) x 10^−2^
CCG_1_G_2_*CG_3_*CC (G_2_G_3_-di)	5.0 (±1.33) x 10^−10^	4.7 (±0.01) x 10^6^	0.2 (±0.02) x 10^−2^

The association rate (k_a_), dissociation rate (k_d_) and binding affinity constant (K_D_) values of the interaction of UvrA with FAAF-modified *Nar*I mono- and di-adducts in the presence of ATP determined by SPR analysis of the interaction of UvrA_2_.

### Electrophoretic mobility shift assay (EMSA)

Binding of XPC to various DNA substrates was analyzed by a gel mobility shift assay as described previously [49]. Typically, DNA substrates (0.5–1 nM) were incubated with varying concentrations of protein at 30°C in 20 *μ*L of binding buffer [20 mM Hepes-KOH, pH 7.9, 75 mM KCl, 5 mM MgCl_2_, 1 mM DTT, 5% glycerol, 100 *μ*g/mL acetylated BSA (Promega)]. Reactions then were placed on ice, 2 *μ*L of 80% (v/v) glycerol was added, and the mixture was immediately loaded onto a 3.5% native polyacrylamide gel and electrophoresed at 80 V in 1× TBE buffer for 2 h at 4°C. The gels were dried and exposed to phosphoimage screens overnight. Quantification of the radioactivity was carried out using a Fuji FLA-5000 scanner with the ImageGuage software.

### Construction of closed-circular plasmid with adducts

The following double-stranded oligonucleotide was cleaved by KpnI and Xba1 (both lowercase) for insertion into the multiple cloning site of the vector pTZ19U between the *XbaI* and *KpnI* restriction sites using the QuikChange II Site-Directed Mutagenesis Kit (Agilent Technologies). The 16-mer sequence containing the *Nar*I hotspot of Fig 1B is underlined.

NER_ins1 5’-CGGggtaccCCGCTCTCGGCGCCATCACTTAGtctagaCTAG-3’

NER_ins2 3’-GCCccatggGGCGAGAGCCGCGGTAGTGAATCagatctGATC-5’

The NER-pTZ19U plasmid was propagated in *E*. *coli* (DH5α) cells, which were infected with virus M13KO7 (NEB) to generate single-stranded plasmid which was purified using the M13 isolation maxi kit (Omega Biotek). Closed-circular double-stranded plasmid containing adduct was made by priming the single-stranded NER-pTZ19U plasmid with 5’-phosphorylated 16-mer oligos (Fig 1B) containing adduct in the presence of Sequenase 2.0 (Affymetrix) and T4 DNA ligase (Promega) following the manufactures protocol. Closed-circular plasmid DNA containing adduct then was purified by agarose gel electrophoresis and elution.

### HeLa whole-cell extract preparation

Whole cell extracts were prepared from HeLa cell pellets purchased from the National Cell Culture Center. The thawed cell pellet was resuspended in four packed-cell volumes (PCV) of 10 mM Tris-HCl pH 8.0, 1 mM EDTA, 5 mM DTT, then incubated on ice for 20 min. The cells were lysed by homogenization in a Dounce homogenizer using eight strokes of the B pestle. Four PCV of 50 mM Tris-HCl pH 8.0, 10 mM MgCl_2_, 2 mM DTT, 25% sucrose (w/v), 50% glycerol (v/v) were added and the mixture was stirred gently. One PCV of saturated (NH_4_)_2_SO_4_ (pH 7.0) was added slowly, then the mixture was stirred for 20 min at 4°C. The lysate was clarified by centrifugation at 11,500xg for 30 min at 4°C. The supernatant was transferred to a fresh tube and solid (NH_4_)_2_SO_4_ (0.33g/ml of suspension) was added. The suspension was mixed for 30 min and 0.01 ml of 1M NaOH per 10 grams of (NH_4_)_2_SO_4_ was added. The precipitated proteins were collected by centrifugation at 11,500xg for 30 min and resuspended in dialysis buffer (25 mM Hepes-KOH pH 7.9, 100 mM KCl, 12 mM MgCl_2_, 0.5 mM EDTA, 2 mM DTT, 12% glycerol) and was dialyzed against the same buffer overnight. Following dialysis the extract was clarified by centrifugation at 10,000xg for 10 min and aliquots of the supernatant were stored at -80°C.

### Dual incision assay

The dual incision assay was adapted from Shivji *et al*. [50]. All incisions were carried out in a total reaction volume of 10 μl. A reaction mixture with 100 μg HeLa whole-cell extract protein in 5x repair buffer [200 mM Hepes-KOH, 25 mM MgCl_2_, 110 mM phosphocreatine (di-Tris salt, Sigma), 10 mM ATP, 2.5 mM DTT and 1.8 mg/ml BSA, (adjusted to pH 7.8), 0.2 μl 2.5 mg/ml creatine phosphokinase (rabbit muscle CPK, Sigma)] was preincubated at 30°C for 10 min. The incision reaction was started with the addition of 50 ng of adduct-containing plasmid DNA, and incubation was continued for another 45 min at 30°C. Samples were placed on ice for 10 min, then 0.5 μl of 1 μM 3’-phosphorylated primer (5’GGGGCAGGTGATGGCGCCGAGAGGGATCCCC-3’) was added and the mixture was heated to 95°C for 5 min and allowed to cool to room temperature for 15 min. Then, a sequenase/[α-^32^P]-dCTP mix was added at 0.25 U of sequenase and 2.5 μCi of [α-^32^P]-dCTP per reaction. The reaction mixture was incubated at 37°C for 3 min before addition of dNTP mix (50 μM dCTP and 100 μM dATP, dGTP, dTTP) followed by an additional 12 min incubation. The reaction was stopped by addition of loading dye and heated to 95°C for 5 min before electrophoresis through a 12% Sequagel (National Diagnostics). Reaction products were visualized with a Fuji Film FLA-5000.

## Results

### Model systems

Mono- and di-FAAF adducted substrates were prepared within the *Nar*I core sequence (5’ -CTCTCG_1_G_2_CG_3_CCATCAC-3’, Fig 1B) as previously reported [33,38,39] and their corresponding duplexes were used to examine their structural and thermodynamic properties (see below). The fluorinated FAAF has been used as a powerful ^19^F NMR probe for investigating arylamine induced conformational heterogeneity [32–34]. This common *Nar*I sequence was ligated to prepare 55 bp substrates for SPR and double-incision assays on closed circular plasmid DNA. Depending on the location of the FAAF, the mono-adducts were designated as *NarI*-G_1_, *Nar*I-G_2_ or *Nar*I-G_3_, in which G_1_, G_2_ and G_3_ signify the position of modified guanine. The di-adducts were designated as *Nar*I-G_1_G_2_, *Nar*I-G_2_G_3_, or *Nar*I-G_1_G_3_, in which the numbers signify the positions of FAAF-modified guanines (Fig 1B). Note, these adducts can exist in the B, S or W conformations (Fig 1C) with the distribution between conformers strongly influenced by the sequence context and nature of other adducts within the *Nar1* sequence [35,51].

### Thermodynamics of mono- and di-FAAF adducts and *E*. *coli* NER

We previously studied the structures and thermodynamics of mono- and di-FAAF duplex adducts derived from the afore-mentioned *Nar*I-16-mer sequence [33,38]. The thermodynamic results summarized in S1 Table revealed that di-adducts produced an additive effect on duplex destabilization relative to the mono-adducts. Briefly, the lesion-induced thermal instabilities (T_m_, S1 Table) were substantially greater for di- (-10.0 ~ -17.9°C) over mono-adducts (-5.3 ~ -8.3°C). Within the di- adducts, the thermal stability was generally in the order of *Nar*I-G_1_G_2_ > *Nar*I-G_1_G_3_ > *Nar*I-G_2_G_3_. Molecular dynamic simulation data indicated that the perturbations of nucleotide base stacking are a major contributor to the observed sequence effect. The di-adducts were more reparable in *E*. *coli* than the corresponding mono-adducts [33,38]. Moreover, we observed a dramatic trend in repair efficiency in *E*. *coli* which parallels the reductions in thermal stability, i.e., *Nar*I-G_2_G_3_ > *Nar*I-G_1_G_3_ >> *Nar*I-G_1_G_2_. Taken together, these results indicate the importance of base stacking and related thermal and thermodynamic instability in the repair of bulky cluster arylamine DNA adducts. However, assessing the overall thermodynamic stability of di-adducts was complicated by the fact that the di-adducts sample a complex range of S/B/W-conformational heterogeneity [38]. The single FAAF adduct at the G_3_ position of the *Nar*I sequence exhibited a preference for the S conformation (61%). Interestingly, greater instability was observed for the G_3_-containing *Nar*I-G_2_G_3_ and *Nar*I-G_1_G_3_ duplexes that produce a higher combined S population (~76 and ~95%, respectively) than *Nar*I-G_1_G_2_ (~49%). In addition, the greater proximity of two FAAF lesions in *Nar*I-G_2_G_3_ (*e*.*g*., just one base apart) compared to that in *Nar*I-G_1_G_3_ (two bases apart) possibly could induce a greater helical distortion.

### Human NER of mono- and di-FAAF adducted DNA

To examine the efficiency of NER in excising the mono- and di-FAAF lesions in the human system, oligonucleotides containing FAAF were generated within the *Nar*I sequence, as described above. These modified oligonucleotides then were incorporated into a closed circular plasmid and subjected to excision with extracts from untreated HeLa cells, which contain the complete NER machinery. A complementary oligonucleotide with a 4-guanine overhang that matches the incision product then was used to generate a radioactive product for visualization and quantification of incision products. Incision experiments were carried out in triplicate under identical conditions, and then the relative incision was measured by separating the incision extension products in a denaturing urea gel as shown in Fig 2. In contrast to the unmodified substrate the modified substrates generated products ranging in size from 25–34 bases.

**Fig 3:**
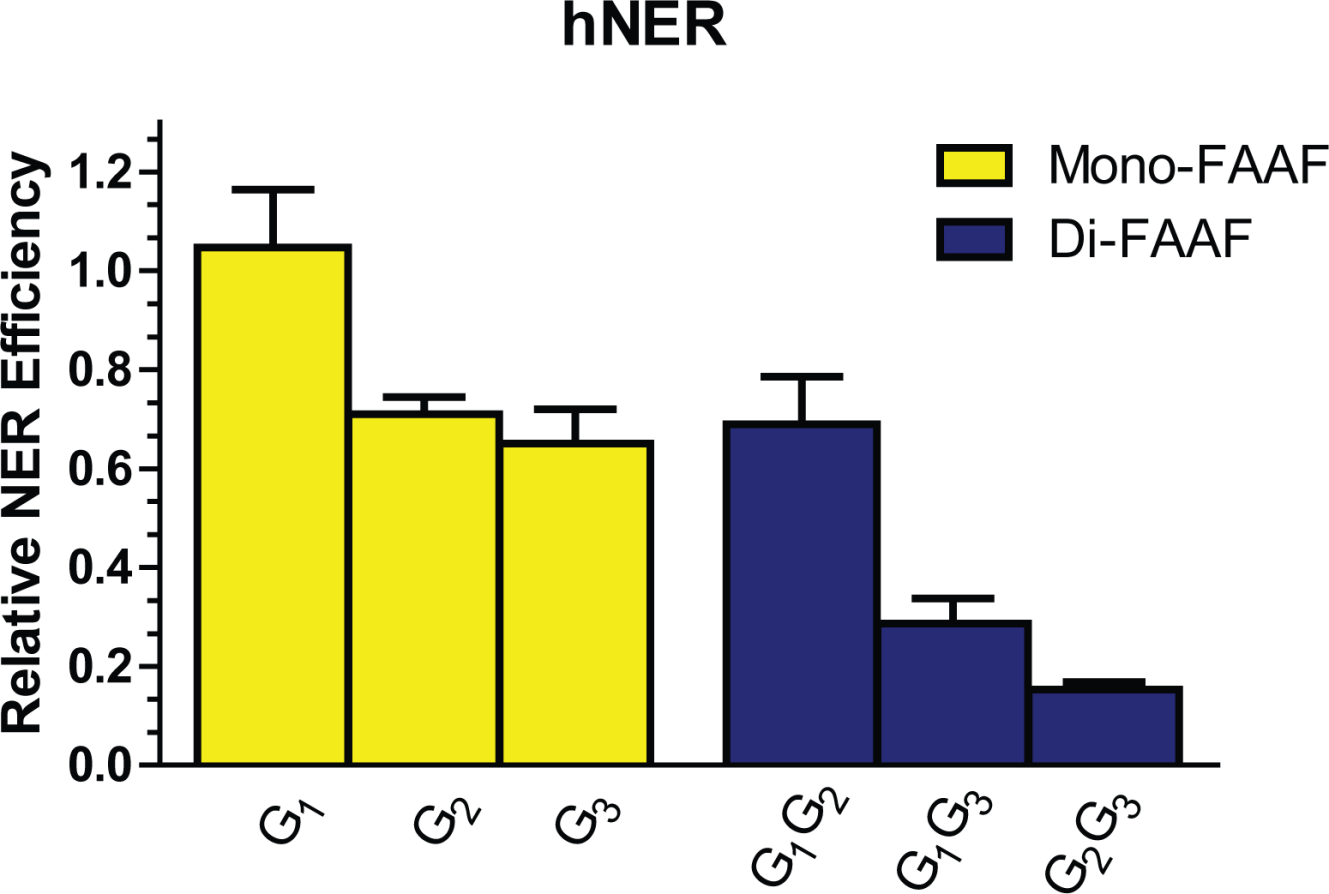
The efficiencies of NER at adducts in the *Nar*I sequence in the human NER system. The relative incision rates of mono-FAAF and di-FAAF adducts in the histogram were calculated by normalizing the mono- (yellow) and di-adducts (blue) relative to the *Nar*I-G_2_G_3_ FAAF value for the *Nar*I-G_1_ FAAF value for the human system. Quantification of NER efficiencies was from at least three independent experiments.

Of the FAAF adducts, *Nar*I G_1_-FAAF was observed to have the maximum incision; therefore, all other *Nar*I adducts were normalized to G_1_ (Fig 3). The relative incision, therefore, was 1 for G_1_, 0.69 for both G_2_ and G_1_G_2_, 0.65 for G_3_, 0.30 for G_1_G_3_, and 0.12 for G_2_G_3_. The observed order for incision efficiency in the mono-adducts was G_1_ > G_2_ ~ G_3_. The relative hNER efficiencies of the di-FAAF adducts in the *Nar*I sequence were G_1_G_2_ > G_1_G_3_ > G_2_G_3_. Previous work on these same mono-adduct substrates has yielded a variety of conclusions [25,27,28]; however, this is the first report to include hNER incision analysis of cluster di-AAF adducts. One interesting observation is that the most thermodynamically stable di-adduct, G_1_G_2_, has no base separating the lesions and, in this respect, is structurally comparable to the UV-induced cyclopyrimidine dimer (CPD) adduct. Work from the Min lab has allowed a better understanding of how XPC binds undamaged and damaged DNA, specifically *via* the CPD adduct [23,24]. Other observations indicate comparability between the cluster di-adduct G_2_G_3_, which has FAAF adducts separated by one intact nucleotide base, and di-nuclear *cis*-platinum complexes [52]. These observations, discussed in further detail below, potentially have significant biological implications.

### *E*. *coli* and human NER produce different incision efficiency patterns on common substrates

Interestingly, there is no direct correlation when comparing the NER incisions between the *E*. *coli* and human systems (Fig 3) [33,38]. Our previous work revealed that in the *E*. *coli* system mono-FAAF adducts were excised in the order of G_3_ ~ G_1_ > G_2_ while di-FAAF adducts were excised in the order of G_2_G_3_ > G_1_G_3_ > G_1_G_2_ [33,38]. In the *E*. *coli* system, the di-adducts overall are more readily incised by the UvrABC system than the mono-adducts; in contrast, in the human system the mono-adducts have significantly greater incision *versus* the di-adducts. Another interesting observation is that the mono- and di-adducts that produced the most incision product in the *E*. *coli* system (G_3_ and G_2_G_3_), led to the least incision product in the human system [33,38].

Although many processes in eukaryotic cells are conserved from prokaryotic systems and operate by similar mechanisms, in the case of NER the issue becomes more complex in that the UvrABC system is made up of only three proteins while in the human system nearly 30 proteins carry out the same function. Also, in humans multiple pathways arose to deal with the repair of a much more complex genome that also exists in a chromatin structure. On the contrary, adducts that destabilize and disorder the DNA are the best substrates for the *E*. *coli* system [33,38] but are poorer substrates in the human system. This leads to the assertion that there are factors other than damage recognition that influences activation of NER in the human system.

### UvrA_2_ and XPC binding to FAAF adducts in the *Nar*I sequence context

**Fig 4:**
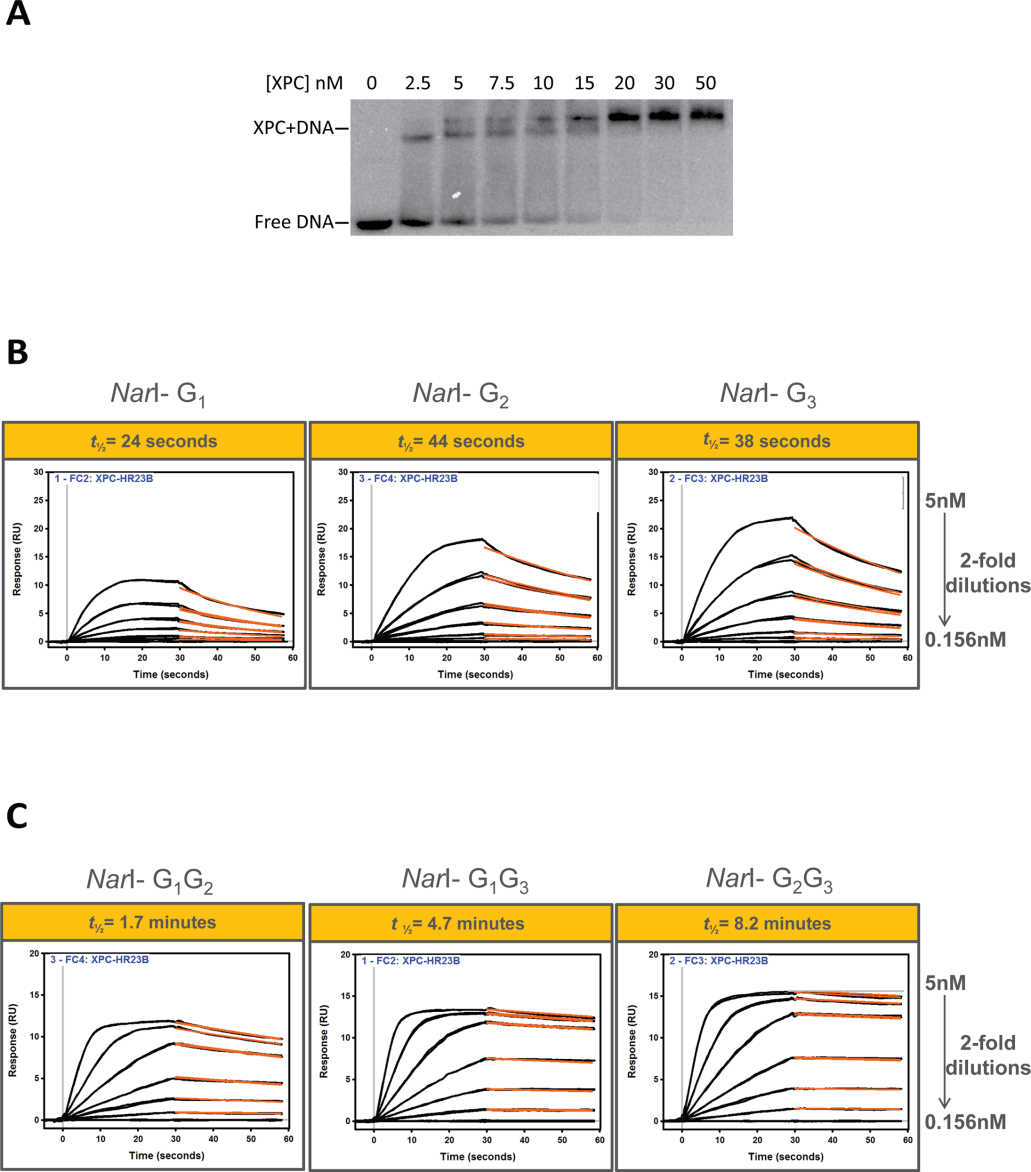
XPC binding to damaged DNA in the *Nar*I sequence. (A) Representative image of XPC binding to *Nar*I-G_1_ in an EMSA assay. XPC protein, at increasing concentration, was incubated with a FAAF-damaged 55-bp oligo. Sensograms showing XPC binding kinetics to mono-FAAF (B) and to di-FAAF adducted substrates (C). SPR responses were recorded for the binding of XPC NER protein (5, 2.5, 1.25, 0.625, 0.313, and 0.156 nM) to FAAF-modified full DNA duplexes. The recorded data are displayed as black lines while red lines represent curve fitting. The half-life (*t*_*1/2*_) is indicated above the curves (in yellow box) and is defined as the time it takes for half of the XPC-DNA complex to dissociate. The fitted curves obtained from fittings using a one-independent site model (“Scrubber”) are displayed (See Methods).

In *E*. *coli* or human NER, UvrA_2_ or XPC is required for the recognition of adduct-induced destabilization of DNA structures as the initial step [9]. The incision efficiency of the UvrABC system on mono- and di-FAAF-adducted DNA has been thoroughly studied in our past work [33,38] and the incision efficiency of the human system on these adducts is presented in the present study. To determine the K_D_, k_a_, and k_d_ of the UvrA_2_, and XPC interactions with the FAAF adducts we employed SPR molecular interaction analysis. By real-time monitoring of UvrA_2_ and XPC binding, a more informed conclusion can be drawn of their binding to and dissociation from adducted DNA. First, as a preliminary test, a traditional method, EMSA, was employed to demonstrate complex formation of XPC and FAAF-adducted DNA (Fig 4A). The gel shift binding pattern is consistent with the previous reports that XPC can bind an adducted oligonucleotide as a multimer at high concentrations, as shown by the two slower migrating bands [25,26]. It was thought that the formation of the slower migrating band could be through biochemical manipulation of the enzyme [25,26]. This EMSA result is of XPC binding to the G_1_ adduct and is representative of the results obtained for all other adducted substrates (data not shown).

Next, mono- and di-FAAF-adducted DNA was used to determine XPC and UvrA_2_ association and dissociation rates by SPR. The SPR binding results are shown in Fig 4 and S2 Fig, in which the average of the triplicate data is displayed. Use of the usual simple 1:1 Langmuir-type binding model did not produce desirable closeness of fit between the protein and modified DNA (S1 Fig). The fitting for XPC binding was improved somewhat (for di-adducts especially) by applying the ‘heterogeneous ligand’ model which is designed to accommodate the interaction of one protein analyte with two ligand sites on the surface. The usual remedy such as reducing the magnitude of the concentration gradient did not significantly mitigate the lack of ideal curve fittings, nor did decreasing the density of the immobilized DNA construct on the surface or increasing the flow rate. It is clear that the adduct-induced conformational heterogeneity of our substrates contributes many non-ideal molecular complexities, e.g., molecular diffusion affects, analyte heterogeneity [53,54], steric hindrance, and protein-DNA binding cooperativity and stoichiometry (*e*.*g*., see EMSA in Fig 4A). These factors affected the kinetic rate constants, which made them less reliable. However, we observed a dramatic difference in equilibrium dissociation constant (K_D_) values with XPC and association and dissociation rates between mono- and di-adducts (Table 1). A similar trend was observed with UvrA (Table 2 and S2 Fig). In the SPR experiments, ATP was added to the binding buffer for UvrA_2_ because ATP appeared to stabilize the dimer. This observation is consistent with the known ATPase activity of UvrA_2_ for adduct binding [55].

The binding of UvrA_2_ and XPC to lesions in the *Nar*I sequence is more stable with the di-FAAF adducts than the mono-FAAF adducts. Importantly, our SPR binding results are similar to previous work demonstrating the binding affinity of XPC to cisplatin-damaged DNA [56,57]. It is of note that the thermostability (ΔT_m_) of these adducts varies and that the di-adducts have the lowest thermostability (G_1_ >G_2_ > G_3_ >> G_1_G_2_ > G_1_G_3_ > G_2_G_3_) (S1 Table). The binding of UvrA_2_ to the di-adducts was observed to be stronger than binding to the mono-adducts (Table 2). This is in agreement with the finding that the NER efficiency in *E*. *coli* is higher for the di-adducts over the mono-adducts. The tightest UvrA_2_-FAAF binding was to G_1_G_3_ where the adducts are separated by two intact nucleotide bases with a dissociation rate of 2.7 x 10^−10^. Interestingly, the correlation between equilibrium constants and NER efficiency for UvrA_2_ and XPC with mono- *versus* di-FAAF-adducted DNA are conversely related. In the case of XPC-FAAF binding, the di-adducts bound with significantly slower dissociation (off) rates relative to the mono-adducts. These results are consistent with conformational changes accompanying the protein-DNA interactions, which could provide the basis for deciphering the binding/repair mechanisms. Comparing K_D_ values (Table 1), the di-FAAF-adducted DNAs show more stable XPC binding than the mono-adducts. The tightest XPC binding was to G_2_G_3_ with a dissociation rate of 0.42 x 10^−11^. The low K_D_ for XPC di-adduct binding signifies a much tighter association of XPC protein to clustered lesions. Table 1 summarizes these findings of XPC binding compared to the thermodynamic stability, the XPC-DNA complex half-life, and the incision efficiencies of FAAF adducts in the human NER system. It should be noted that the G_1_G_2_ adduct has a longer half-life but similar incision to the mono-adducts, G_1_ and G_3_. Interestingly, this is the only cluster adduct studied in which the adducts are on adjacent nucleotide bases and its binding half-life is much shorter than those of G_1_G_3_ and G_2_G_3_ with the di-adducts separated by at least one intact nucleotide bases.

**Fig 5:**
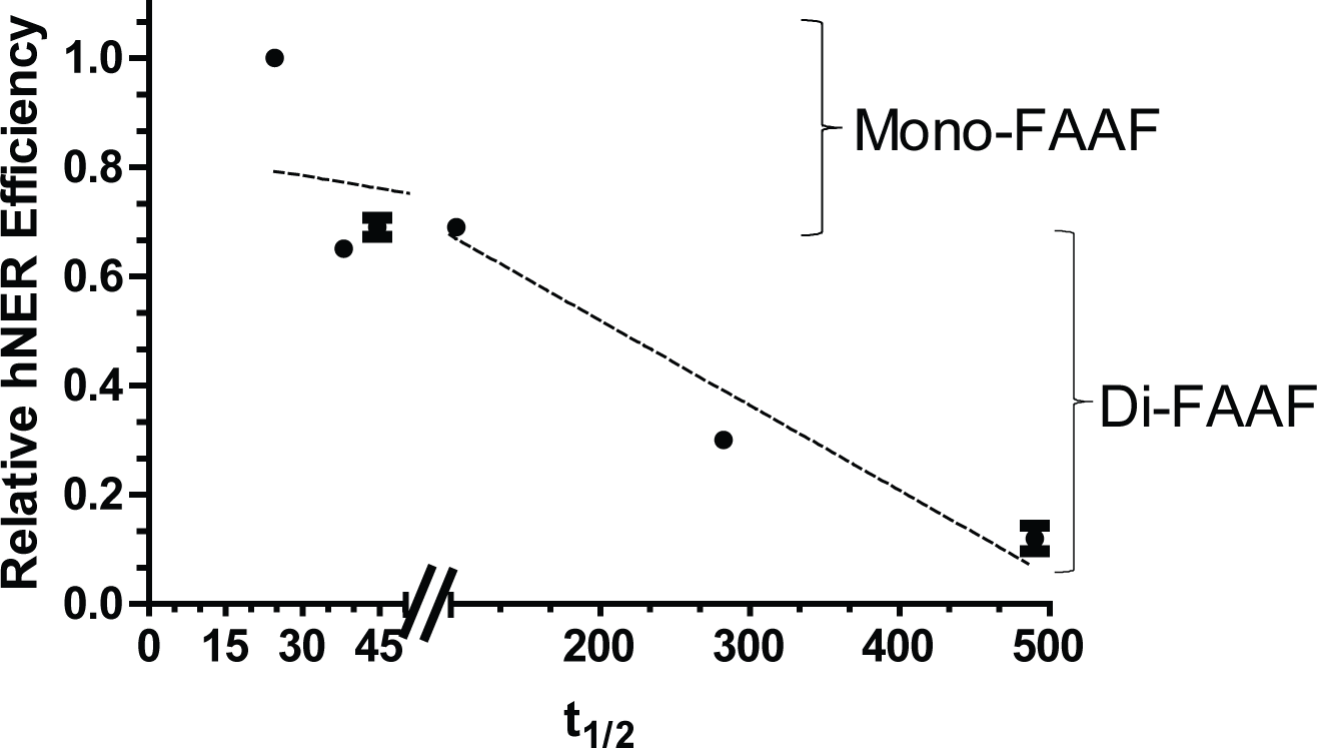
Comparison of hNER efficiency and half-life (*t*_1/2_) of the XPC-DNA complex. The data points were analyzed independently as mono- or di-FAAF adduct groups and the two dashed lines indicates the group trends. Mono- and di-adducts are indicated on the right.

By calculating the half-life we observed that XPC-DNA complexes containing the di-FAAF adducts were on average at least 8 times more stable than complexes with the mono-FAAF adducts (Fig 4B and 4C, Table 1). In the extreme case the half-life of XPC-DNA complexes containing *Nar*I di-G_2_G_3_-FAAF was 20 times longer than that of *Nar*I mono-G_1_-FAAF. The half-life demonstrates an inverse relationship with the hNER efficiency (Fig 5). This indicates that the longer XPC stays bound to the adduct site, the less productive the human NER process. These data are provocative as they suggest that a strong DNA damage recognition or binding itself does not necessarily guarantee efficient NER.

## Discussion

Various studies have attempted to relate the protein binding interactions involved in DNA damage recognition with NER excision efficiency utilizing a wide array of DNA damages [26,58]. This work proposes a novel mechanism beyond the conventional concept that the binding capability of DNA damage recognition proteins directly relates to the ability of the repair process to remove the damage from DNA. The presented SPR/hNER results suggest strongly that di-FAAF adducts fail to produce a productive complex for hNER even though the damage recognition binding is strong. In other words, robust XPC binding (K_D_ of 10^−9^ ~ 10^−10^ M) may be required for initiation of hNER. However, unusually strong XPC binding (K_D_ <10^−11^) and, more importantly, the extremely slow dissociation of di-FAAF adducts, and thus the long residence time (*t*_*1/2*_) of XPC at the damage site, could be detrimental to recruitment of subsequent downstream proteins to complete the hNER process (Fig 5). We demonstrate that in addition to the equilibrium binding affinity of XPC for DNA damage, kinetics and the off-rate of the interaction also play critical roles in determining the NER efficiency. This is particularly true for certain types of DNA adducts, such as the di-FAAF examined here, which have a long XPC residence time during DNA damage recognition. Since dissociation of XPC from the damage site after initial recognition is necessary for subsequent binding of other repair factors in the mechanism of NER [15,58,59], it is possible that such an extended residence of XPC at the damage site would likely make dissociation the rate-limiting step of NER. This could lead to inefficient DNA repair even though the XPC-damaged DNA binding affinity is high. Our findings may help us better understand the complex mechanisms relating protein binding and adduct clearance *via* NER.

For the mono-FAAF adducts we observed that the repair efficiency of the G_1_ adduct was significantly higher than that of the G_2_ and G_3_ adducts, in agreement with the shorter half-life of XPC on DNA adducted at the G_1_ rather than the G_2_ or G_3_ positions (Table 1). This contrasts with different reported preferences for repair in HeLa cell extracts of adducts at the G_2_ position [27,28] or at the G_3_ position [25]. These differences in NER preferences may stem from the nature of the DNA constructs used in the respective studies. The long-range sequence context of the DNA in which the adducted *Nar*I site is embedded differs significantly between our construct and that of Yeo *et al*. as do the plasmids carrying these constructs (pTZ19U vs. pBluescript II SK+, respectively) [25]. In addition, the studies reporting a preference for repair of adducts at the G_2_ position employed relatively short (<150 bp), linear, and internally labeled constructs that may display different torsional stresses on the DNA double helix in the HeLa extracts. Most studies on sequence context on NER efficiencies focus on the position effects within the *Nar*I sequence rather than the influence of the long-range sequence in which it is embedded or the effect of super-helical stresses within the circular plasmids. Further studies are needed to resolve the influence of these experimental parameters on the NER efficiencies. However, the SPR, thermodynamic, EMSA and repair efficiency data reported here are internally consistent in highlighting the importance of the half-life of the XPC-DNA complex in determining NER efficiency.

Our finding that the increased residence time of XPC on damaged DNA containing di-adducts reduces repair efficiency is consistent with the *in vivo* studies of NER protein binding dynamics to chromatin and at damaged DNA sites, especially for XPC [60]. However, we analyzed reconstituted systems of short adducted dsDNAs of non-UV induced DNA damage plus purified XPC-RAD23B or adducted plasmid DNA plus a fractionated extract from HeLa cells not exposed to UV. Thus, our data may not directly correlate to cellular studies showing an observed influence of DDB2 (damaged DNA-binding 2) protein. This is also possibly true for the effects of the ubiquitin-dependent p97 segregase and centrin-2 on XPC binding/release dynamics from UV-damaged chromatin DNA [61]. Further studies are needed to test how such cellular factors might reduce the binding and retention time of XPC on DNA containing mono- and di-FAAF adducts, and to extend these analyses to UV-induced damage. However, our data are consistent with their observation that the prolonged retention of XPC in chromatin or an increase t_1/2_ reduces the NER efficiency [61], though the prolonged retention in the previous UV-induced damage study was due to the deficiency of p97 segregase.

The *E*. *coli* NER system has been studied for decades, leading to many breakthroughs in our understanding of how cells can repair DNA damage. These studies have provided vital information that is applicable to our understanding of the human NER system; however, these two systems are not directly comparable when considering damage recognition. Tight binding of recognition proteins may imply a better incision substrate, and this is true for *E*. *coli*, but in the human system much tighter binding can lead to a longer residence time, which results in a decrease in substrate processing. Analysis of previously reported structures of XPC- and UvrA_2_B (or UvrA_2_B_2_)-damaged DNA interactions may provide some understanding of our observed differences of the FAAF adduct incisions between hNER and UvrABC systems [62–64]. In both *E*. *coli* and human systems a damage recognition protein is required to initiate the NER. UvrA_2_B and XPC-RAD23B, respectively, fill this role in the GGR sub-pathway of NER. Interestingly, although both protein complexes recognize DNA damage, dissociation of these proteins from the damage site after recognition is quite different. In the case of UvrABC, UvrA_2_ dissociates, while UvrB remains bound to the damage. In contrast, in hNER, RAD23B dissociates shortly after binding; however, XPC remains bound [65]. Interestingly, structural evidence revealed that UvrA_2_ makes contact with the DNA that flanks the damage site and has no contact with the lesion itself [64], while UvrB establishes lesion contact, utilizing a β-hairpin domain to insert into the DNA strands and flip-out bases opposing the lesion [63,66]. On the other hand, XPC appears to be responsible for both roles carried out by UvrA_2_ and UvrB of the UvrA_2_B complex since XPC also inserts its β-hairpin domain between DNA strands at the damage site [23,24]. The β-hairpin insertion is likely to make protein-DNA interaction more stable and, thus, the protein less likely to dissociate from DNA. Since UvrA_2_ does not carry out β-hairpin insertion while XPC does, it is possible that UvrA_2_ would be energetically easier to dissociate from DNA than XPC whose dissociation is more damage-type dependent. Thus, the type of di-FAAF adduct may increase the affinity of XPC at the damage site, leading to a longer residence time. For mono-FAAF adducts, in contrast, a normal *t*_*1/2*_ keeps the dissociation from being a rate-limiting step, remaining close to the regular binding-repair efficiency correlation. Our data supports the notion that XPC has an increased residence time at clustered lesion sites, making the dissociation from the lesion the rate-limiting step.

**Fig 6:**
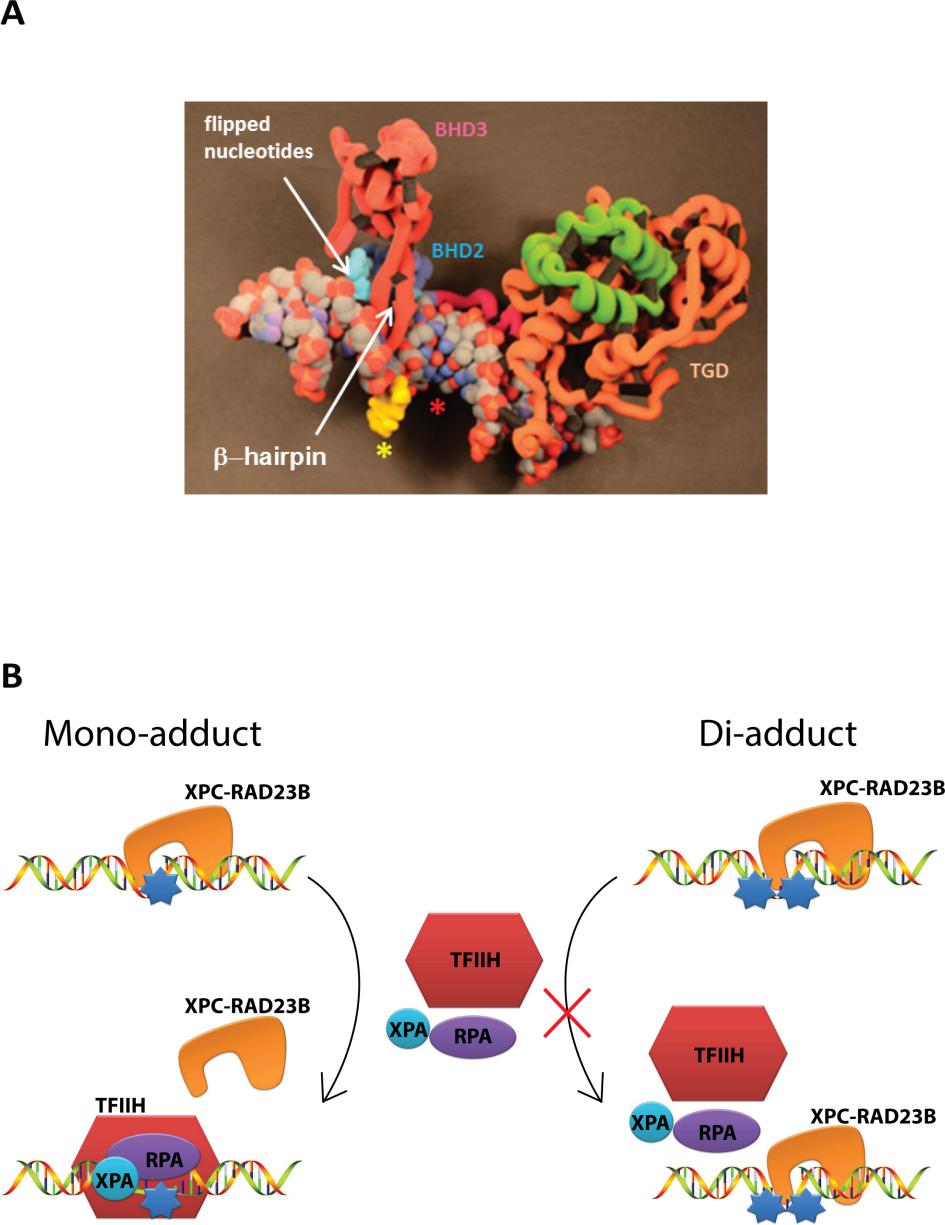
Proposed model for XPC interaction with DNA-adduct site. (A) 3D-printed model (not simulated) to illustrate the potential binding of the yeast XPC-RAD23B ortholog, Rad4/Rad23, to the mono-G_1_-FAAF duplex based on PDB ID 2QSG. The β-hairpin domains (BHD2 and BHD3) and the transglutaminase-homology domain (TGD), which are involved in protein-DNA interaction, are indicated. The domains were adapted from previous crystal structure analysis by Min *et al*. [24]. The duplex sequence used in this model is identical with that of Min’s crystal work except that the CPD lesion was replaced by FAAF-G_1_ (yellow *, as shown). The site of additional FAAF in the di-G_2_G_3_-adduct is designated in red asterisk. The insertion of the BHD3 β-hairpin was accompanied with flipping of the mismatched bases (cyan) on the complimentary sequence. (B) A schematic illustrating the proposed mechanism of action where XPC is loosely bound to mono-FAAF adducted DNA (left) or tightly bound to di-FAAF adducted DNA (right). Following dissociation of XPC from the damage site subsequent NER factors are recruited to complete the excision of the damaged base; however, in the di-adduct situation XPC is retained on the damaged DNA, delaying successful NER completion.

Fig 6A shows a ‘hypothetical’ 3D model of the G_1_ mono-FAAF adduct that was printed (not simulated), derived from the published Rad4-CPD DNA structure (PDB ID# 2QSG) [24]. The G_1_ mono-FAAF and G_2_G_3_ di-FAAF (not shown, additional FAAF site designated as red*) adducts exhibit distinctive differences in hNER (1 *vs*. 0.12 relative efficiencies) (Fig 3) and XPC residence time (24 *vs*. 495 s) (Table 1), as well as DNA thermal stability (Δ*T*_m_ -5.3 *vs*. -17.9°C) (S1 Table). The CPD mismatch site was simply replaced by FAAF (yellow,*) for visualization of the G_1_ mono-FAAF. The mono-lesion is expected to produce a similar NER complex as the CPD; that is, Rad4 inserts a β-hairpin into the damaged site with most of the modified strand being fully exposed and the mismatched bases (cyan) on the complimentary strand flipped out the double helix. The di-G_2_G_3_ adduct, however, contains two FAAF adducts on separated by one intact nucleotide base and is likely to exhibit very different conformations that are responsible for its unusually strong binding and slow dissociation (Table 1). Possibilities include additional DNA interactions with the BHD3/BHD2 and TGD protein segments of XPC protein; the latter have been considered to be responsible for the highly kinked DNA conformations. These additional XPC-lesion interactions could provoke conformational alterations, which may affect the logistics of the subsequent verification step. One potential future study is to crystalize Rad4 or XPC complexed with the afore-mentioned mono-G_1_ and di-G_2_G_3_-FAAF adducts used in this study. If successful, the results are likely to provide valuable structural insights on XPC-DNA interactions that contribute to the large discrepancy in their residence time and reveal important clues regarding the structural requirements for recruitment of other NER proteins and subsequent lesion verification.

Tight binding during damage recognition may imply a better substrate for incisions, and this is true for *E*. *coli* NER, but in the human system binding too tightly can lead to a longer residence time, which decreases DNA repair (Fig 6B). In addition to the new insights into understanding the mechanisms of hNER, the inverse relationship between the *t*_*1/2*_ of tight binding and NER efficiency suggests a novel strategy as a new therapeutic approach in cancer therapy. Given that DNA damaging agents have been widely exploited for anticancer activities, targeting properly spaced di-adducts or a cluster-like drug that effectively stalls XPC or other damage recognition proteins could lead to strong resistance to repair and, thus, a higher efficiency in killing cancer cells. Recent studies have introduced new models that utilize residence time in drug design and to increase the efficacy of known drugs [67,68]. These models could be applied to our system allowing for design of novel drugs that could increase the residence time of XPC on damaged DNA for cancer therapeutics.

In summary, the present study suggests that dissociation of XPC from adducted DNA is the determining factor for successful NER elimination of adducts. In recognizing the types of adducts that are comparable to *Nar*1-G_2_G_3_ and *Nar*I-G_1_G_3_ cluster adducts, XPC can be stalled on these damage sites, preventing clearance of induced adducts. Exploiting the high proliferative rate of cancer cells and the slow dissociation rate of XPC from clustered adducts allows for a more efficiently targeted approach to cancer therapy. Furthermore, the current work also advances our understanding of the intricacies of the NER mechanism.

## Supporting Information

S1 Fig Variation of curve fitting between the heterogeneous ligand and langmuir fitting models. Representative SPR sensograms of mono- (A) and di-adducted (B) duplexes demonstrating the variation of curve fitting using the heterogeneous ligand fitting model (top) and the 1:1 Langmuir fitting model (bottom). The XPC protein concentrations used were 2.5, 1.25, 0.62, 0.31, and 0.15 nM.
(TIF)

S2 Fig The effects of ATP on the UvrA binding to FAAF-damaged DNA in the *Nar*I sequence. Sensograms showing UvrA binding kinetics to mono-adducted substrates in the absence of ATP (A) or in the presence of ATP (B) and to di-adducted substrates in the absence of ATP (C) or in the presence of ATP (D). SPR responses were recorded to of the binding of UvrA NER protein (250, 125, 62.5, 31.2, 15.6, and 7.8 nM) to modified full DNA duplexes. The recorded data are displayed as black lines while red lines represent curve fitting. The fitting curves obtained from fittings using a one-independent site model are displayed.
(TIF)

S3 Fig Representative MALDI-TOF mass spectra. MALDI-TOF mass spectra analysis of unmodified (orange) or FAAF-modified (red) substrates (55-mer).
(TIF)

S1 Table Thermal and thermodynamic parameters of mono- and di-FAAF modified *Nar*I duplexes. Comparative thermodynamic parameters are listed for the FAAF-modified substrates. This is a summary of previously reported data for mono- and di-FAAF substrates [33,38]. The average standard deviations for − ΔΔH, − ΔΔG, and ΔΔT_m_ are ±3.0, ±0.4, and ±4.0, respectively [33,38]. ΔΔH = ΔH(modified duplex)– ΔH (control duplex). ΔΔG = ΔG (modified duplex)– ΔG (control duplex). ΔΔT_m_ = ΔT_m_ (modified duplex)– ΔT_m_ (control duplex).
(TIF)
