# Supplementary figures and images for: Dissociation Dynamics of XPC-RAD23B from Damaged DNA Is a Determining Factor of NER Efficiency
Source: PLoS One. 2016 Jun 21;11(6):e0157784. doi: 10.1371/journal.pone.0157784 (PMC4915676; doi:10.1371/journal.pone.0157784)

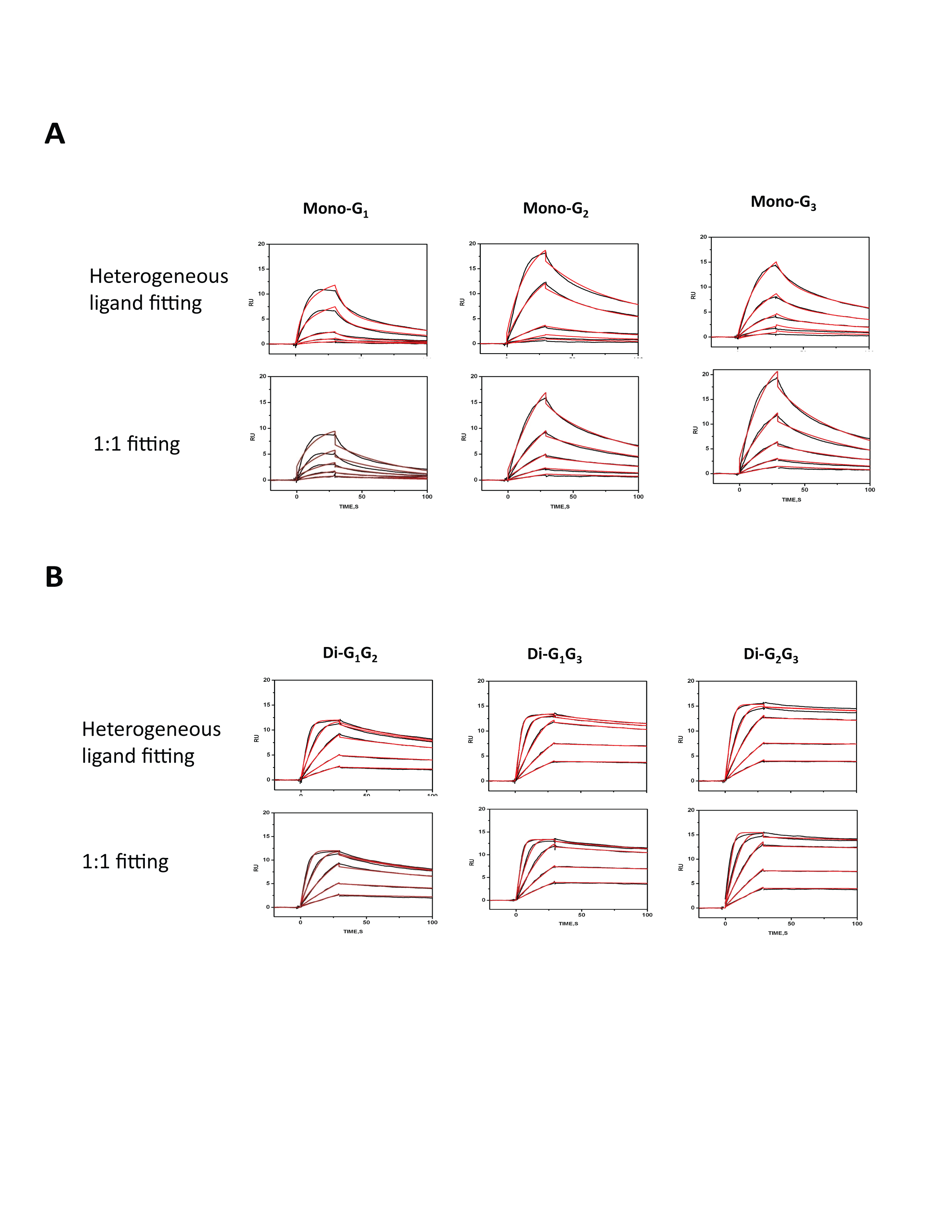

Supplement: S1 Fig — Representative SPR sensograms of mono- (A) and di-adducted (B) duplexes demonstrating the variation of curve fitting using the heterogeneous ligand fitting model (top) and the 1:1 Langmuir fitting model (bottom). The XPC protein concentrations used were 2.5, 1.25, 0.62, 0.31, and 0.15 nM. (TIF) [file pone.0157784.s001.tif]

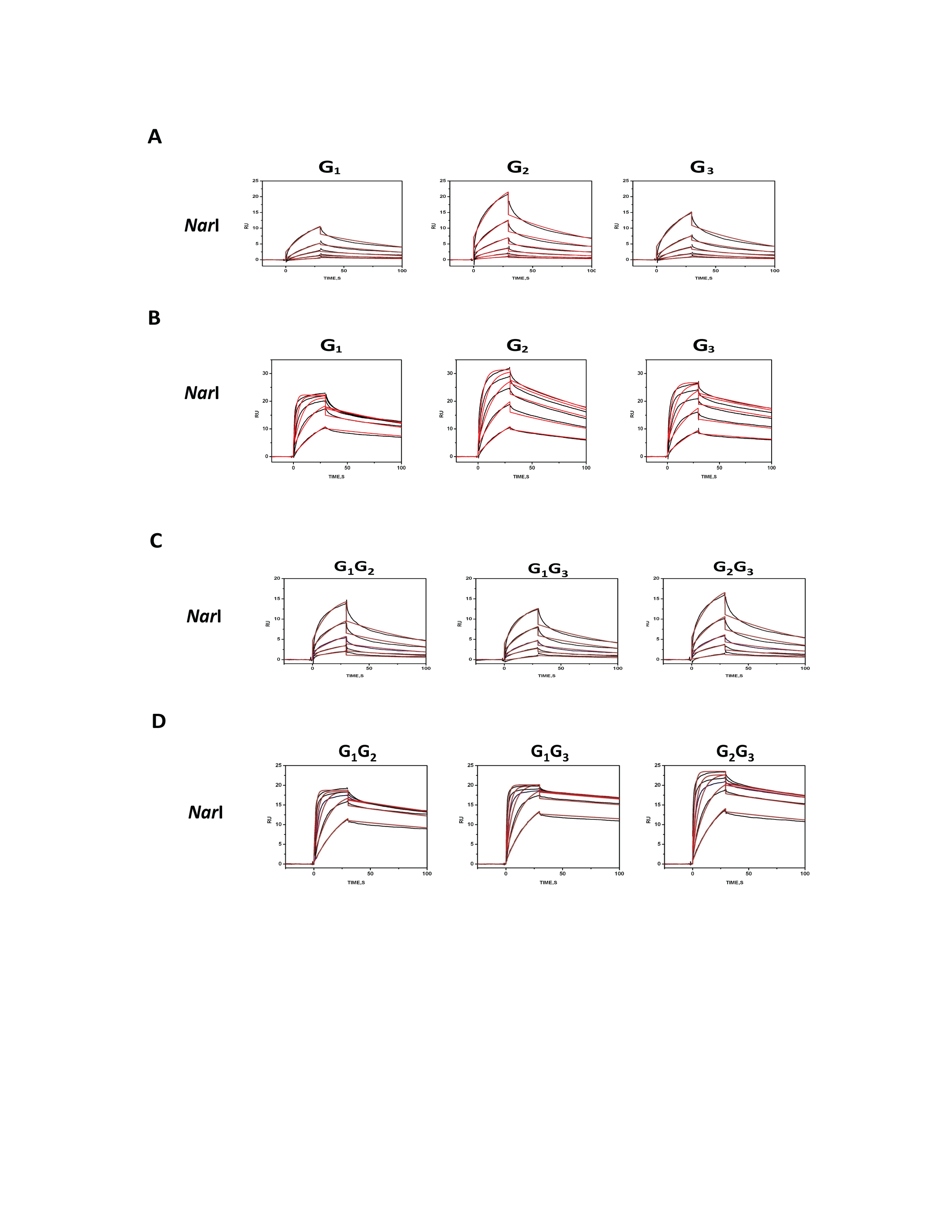

Supplement: S2 Fig — Sensograms showing UvrA binding kinetics to mono-adducted substrates in the absence of ATP (A) or in the presence of ATP (B) and to di-adducted substrates in the absence of ATP (C) or in the presence of ATP (D). SPR responses were recorded to of the binding of UvrA NER protein (250, 125, 62.5, 31.2, 15.6, and 7.8 nM) to modified full DNA duplexes. The recorded data are displayed as black lines while red lines represent curve fitting. The fitting curves obtained from fittings using a one-independent site model are displayed. (TIF) [file pone.0157784.s002.tif]

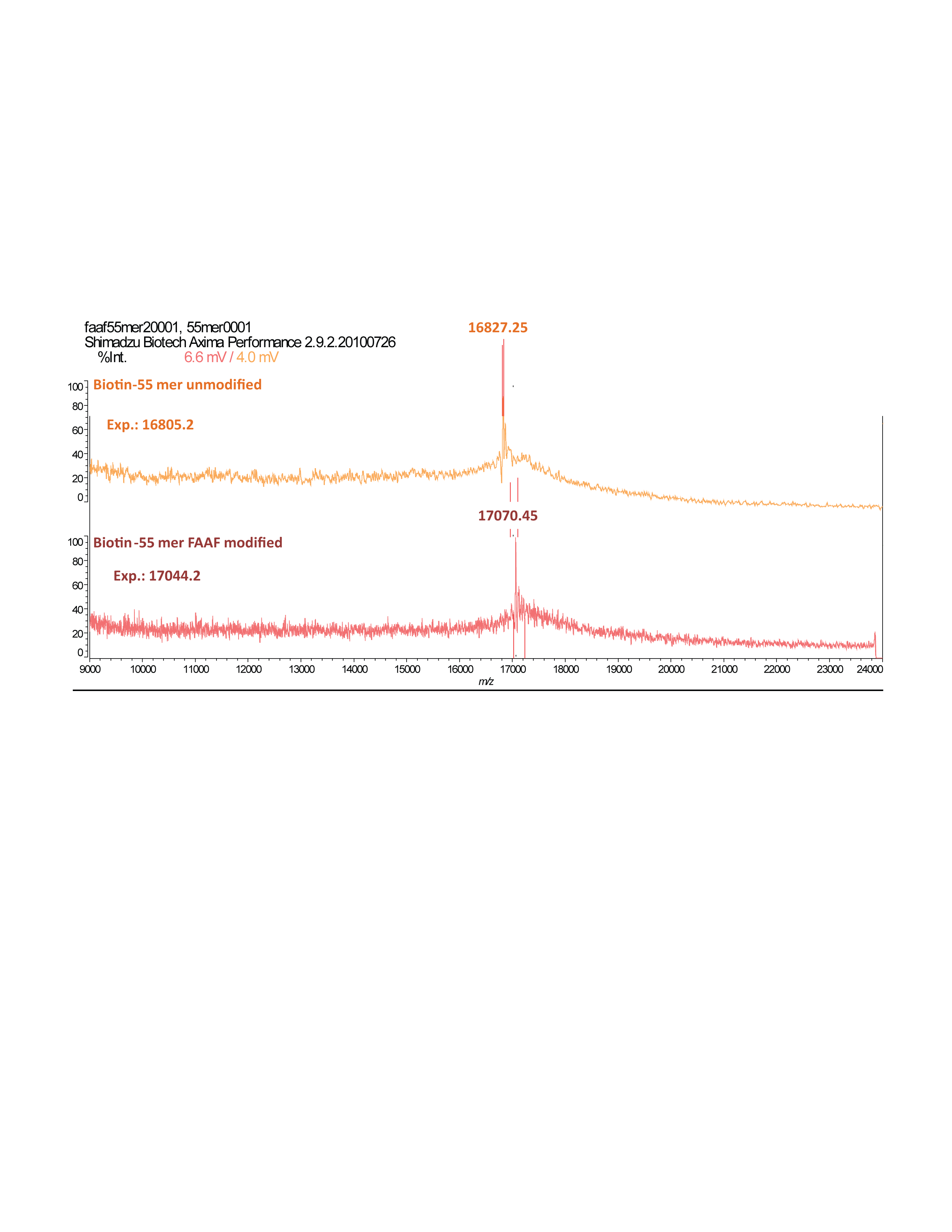

Supplement: S3 Fig — MALDI-TOF mass spectra analysis of unmodified (orange) or FAAF-modified (red) substrates (55-mer). (TIF) [file pone.0157784.s003.tif]

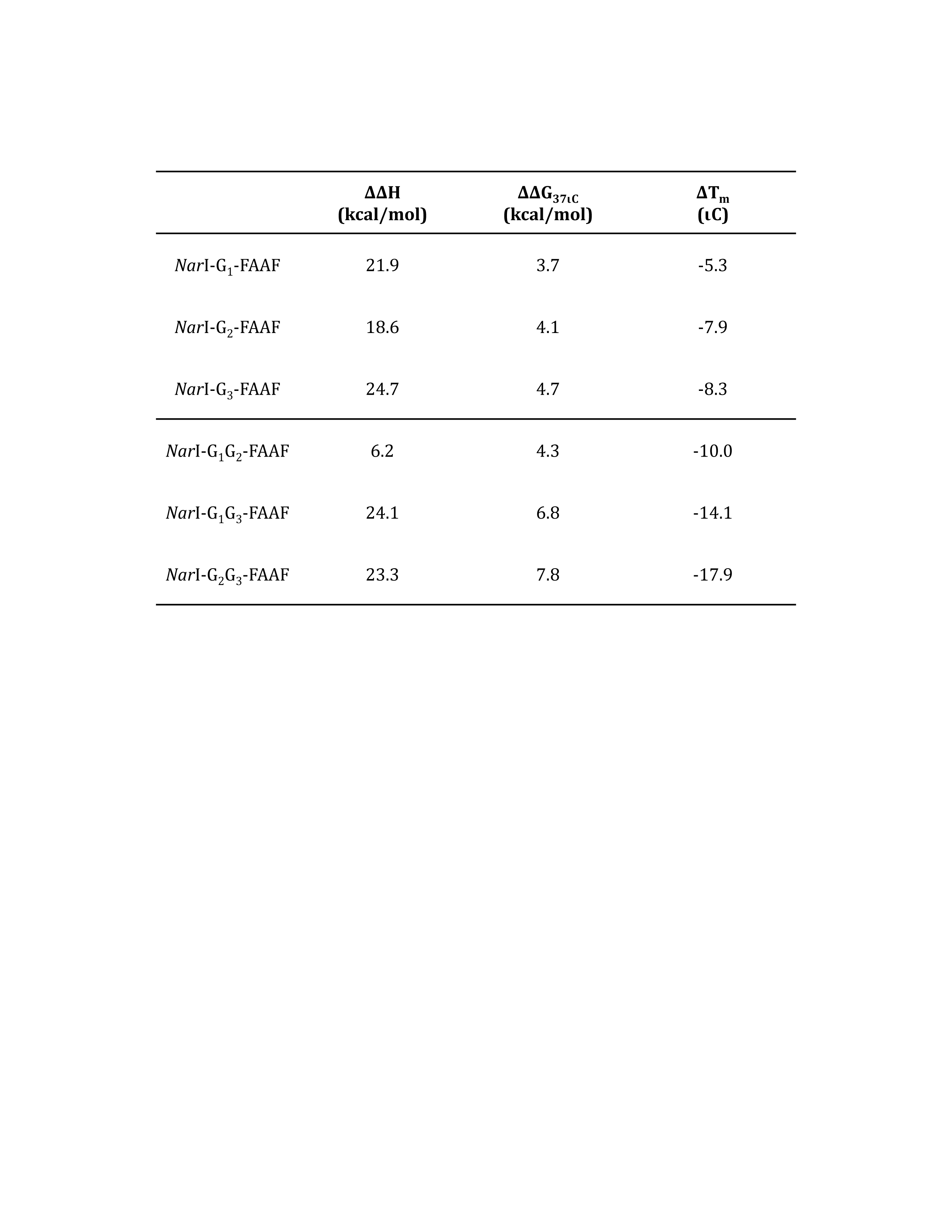

Supplement: S1 Table — Comparative thermodynamic parameters are listed for the FAAF-modified substrates. This is a summary of previously reported data for mono- and di-FAAF substrates [33,38]. The average standard deviations for − ΔΔH, − ΔΔG, and ΔΔTm are ±3.0, ±0.4, and ±4.0, respectively [33,38]. ΔΔH = ΔH(modified duplex)– ΔH (control duplex). ΔΔG = ΔG (modified duplex)– ΔG (control duplex). ΔΔTm = ΔTm (modified duplex)– ΔTm (control duplex). (TIF) [file pone.0157784.s004.tif]
